# Influence of the tryptophan-indole-IFNγ axis on human genital *Chlamydia trachomatis* infection: role of vaginal co-infections

**DOI:** 10.3389/fcimb.2014.00072

**Published:** 2014-06-03

**Authors:** Ashok Aiyar, Alison J. Quayle, Lyndsey R. Buckner, Shardulendra P. Sherchand, Theresa L. Chang, Arnold H. Zea, David H. Martin, Robert J. Belland

**Affiliations:** ^1^Department of Microbiology, Immunology, and Parasitology, Louisiana State University Health Sciences CenterNew Orleans, LA, USA; ^2^Department of Microbiology and Molecular Genetics, Public Health Research Institute Center, New Jersey Medical School—Rutgers, The State University of New JerseyNewark, NJ, USA; ^3^Section of Infectious Diseases, Department of Medicine, Louisiana State University Health Sciences CenterNew Orleans, LA, USA; ^4^Department of Microbiology, Immunology, and Biochemistry, University of Tennessee Health Sciences CenterMemphis, TN, USA

**Keywords:** *Chlamydia trachomatis*, IFNγ, IDO1, tryptophan, persistence, indole, bacterial vaginosis, vaginal microbiome

## Abstract

The natural history of genital *Chlamydia trachomatis* infections can vary widely; infections can spontaneously resolve but can also last from months to years, potentially progressing to cause significant pathology. The host and bacterial factors underlying this wide variation are not completely understood, but emphasize the bacterium's capacity to evade/adapt to the genital immune response, and/or exploit local environmental conditions to survive this immune response. IFNγ is considered to be a primary host protective cytokine against endocervical *C.* *trachomatis* infections. IFNγ acts by inducing the host enzyme indoleamine 2,3-dioxgenase, which catabolizes tryptophan, thereby depriving the bacterium of this essential amino acid. *In vitro* studies have revealed that tryptophan deprivation causes *Chlamydia* to enter a viable but non-infectious growth pattern that is termed a persistent growth form, characterized by a unique morphology and gene expression pattern. Provision of tryptophan can reactivate the bacterium to the normal developmental cycle. There is a significant difference in the capacity of ocular and genital *C. trachomatis* serovars to counter tryptophan deprivation. The latter uniquely encode a functional tryptophan synthase to synthesize tryptophan via indole salvage, should indole be available in the infection microenvironment. *In vitro* studies have confirmed the capacity of indole to mitigate the effects of IFNγ; it has been suggested that a perturbed vaginal microbiome may provide a source of indole *in vivo*. Consistent with this hypothesis, the microbiome associated with bacterial vaginosis includes species that encode a tryptophanase to produce indole. In this review, we discuss the natural history of genital chlamydial infections, morphological and molecular changes imposed by IFNγ on *Chlamydia*, and finally, the microenvironmental conditions associated with vaginal co-infections that can ameliorate the effects of IFNγ on *C. trachomatis*.

## Introduction

*Chlamydia trachomatis* is an obligate intracellular bacterium that has a unique biphasic developmental cycle. *C. trachomatis* serovars D through K are tropic for columnar epithelial cells of the urogenital tract; the endocervix is the most common site of infection in women, but organisms can ascend into the uterus and Fallopian tubes where they can cause pelvic inflammatory disease (PID) and the longer-term sequelae of tubal infertility and ectopic pregnancy. Infected, untreated women can also vertically transmit their infection to neonates, with consequences including pneumonia. Additionally, infected women have a significantly increased risk of acquiring, shedding and/or transmitting, human immunodeficiency virus-1 (HIV-1) (Wasserheit, 1992; Ghys et al., 1997; Fleming and Wasserheit, 1999; Chesson and Pinkerton, 2000; Farley et al., 2003). Despite extensive public health interventions including education, screening, and antibiotic treatment, *C. trachomatis* infections remain a significant global health problem, with reported U.S. cases reaching 1 million in 2006 and continuing to rise since (Centers for Disease Control, 2011). This burden of cases, together with the predominantly asymptomatic nature of the disease, have led the *C. trachomatis* infection to be called the “silent epidemic” (Wallis, 2001). The development of a vaccine is now considered *a priori* in the control of this infection.

The natural history of genital *C. trachomatis* infection can vary considerably. Untreated infections can be asymptomatic for substantial periods of time, progress to cause significant pathology, or spontaneously resolve without antibiotic treatment (Parks et al., 1997; Golden et al., 2000; Joyner et al., 2002; Morre et al., 2002; Molano et al., 2005; Geisler, 2010; Centers for Disease Control, 2011). It is not understood why some *C. trachomatis* infections can last from months to years; clearly, the organism has the ability to survive by exploiting, adapting to, or evading, certain genital immune and environmental conditions (Brunham and Rey-Ladino, 2005). Establishing the local genital environments that enable, enhance, or deter *C. trachomatis* survival should allow us to: (a) identify the women at risk for extended infections; (b) define the conditions needed for the genital immune system to resolve infection naturally, and (c) aid in designing targeted vaccination and therapeutic strategies. Pertinent to this, *C. trachomatis* is a tryptophan auxotroph; therefore, induction of the tryptophan-catabolizing enzyme, indoleamine-2,3-dioxygenase 1 (IDO1), by the cytokine interferon gamma (IFNγ) restricts chlamydial growth and development in human epithelial cells (Shemer and Sarov, 1985; Byrne et al., 1986, 1989; Carlin et al., 1989; Beatty et al., 1994a; Brunham and Rey-Ladino, 2005). For this reason, IFNγ is considered to be a major anti-chlamydial effector cytokine. *In vitro*, exposure of *C. trachomatis-*infected epithelial cells to IFNγ can result in bacterial death, or, can cause the organism to adopt a viable but non-infectious growth mode that is termed a persistent or abnormal growth form (reviewed by Wyrick, 2010). *In vitro,* dependent on the culture conditions, persistent growth forms can proceed to clearance upon prolonged starvation, or they can reactivate and replicate to produce infectious elementary bodies (EBs) (Byrne et al., 1986, 1989; Beatty et al., 1993, 1994a). Pertinent to IFNγ-induced tryptophan starvation, genital, but not ocular, serovars of *C. trachomatis* have retained a functional tryptophan synthase that enables them to synthesize tryptophan via indole salvage (Fehlner-Gardiner et al., 2002; Caldwell et al., 2003). Thus, if indole is present at a sufficient concentration in the infection microenvironment, genital serovars could circumvent the bactericidal/bacteriostatic effects of IFNγ (Beatty et al., 1993; Fehlner-Gardiner et al., 2002; Belland et al., 2003; Caldwell et al., 2003). This suggests the capacity to synthesize tryptophan in an IFNγ-rich infection microenvironment is an important virulence factor for genital *C. trachomatis* serovars. *In vivo*, the source of indole in the infection microenvironment remains unknown. However, perturbations in the vaginal flora that increase the prevalence of indole-producing bacteria during bacterial vaginosis (BV) may increase the susceptibility of women to extended infections even in the face of a robust IFNγ response, a hypothesis originally proposed by Caldwell and co-workers in a seminal Journal of Clinical Investigation study in 2003 (Caldwell et al., 2003). However, until recent work by others and us, the nature of a “real” clinical infection, the *in vivo* growth characteristics of *C. trachomatis*, and the composition of the genital milieu *in vivo* have remained almost completely uncharacterized, precluding a test of this hypothesis. The purpose of this review is to: (a) describe the recent advances made in the characterization of the normal and perturbed vaginal microbiome that are pertinent to the indole-rescue hypothesis; (b) describe recent *in vivo* data to support and extend this hypothesis, including evidence that *C. trachomatis* can adopt a persistent growth mode *in vivo*, and that BV provides an indole-rich genital environment; and (c) explicate the mechanism by which BV and/or the vaginal *Trichomonas vaginalis* (TV) co-infections could modulate the effect of IFNγ on *C. trachomatis* growth and clearance *in vivo*, including ramifications on clinical outcomes and choice of treatment.

## IFNγ-mediated immunity to genital *C. trachomatis* infection

Several lines of evidence indicate genital *C. trachomatis* infection induces human immunity and that this immunity is variably protective, as reviewed recently (Batteiger et al., 2010). These include: (a) young age as a significant risk factor for infection acquisition (Arno et al., 1994); (b) reduced organism load with repeat infections (Barnes et al., 1990); (c) natural history studies documenting spontaneous resolution of infection (Parks et al., 1997; Golden et al., 2000; Joyner et al., 2002; Morre et al., 2002; Molano et al., 2005; Centers for Disease Control, 2010; Geisler, 2010); and (d) association of spontaneous resolution of infection with subsequent protection from incident disease (Geisler et al., 2013). Immunity in humans appears to develop slowly and protection from infection is generally thought to be robust only after multiple exposures and can be as short as several months (Katz et al., 1987; Molano et al., 2005), a finding corroborated in animal models (Rank and Whittum-Hudson, 2010). Further, chronic infection has been associated with pathology and the long-term sequelae of disease. Thus, significant current declines in PID are attributed to the aggressive “seek and treat” public health intervention strategies that are now in place in many developed countries (Brunham and Rekart, 2008, 2009). Paradoxically, this strategy has also been partially attributed to increasing rates of disease by blunting the development of protective immunity to this pathogen, thereby increasing the susceptibility of the population to disease (Brunham and Rekart, 2008). This has been termed the “arrested immunity” hypothesis and is corroborated by antibiotic intervention studies in mice (Su et al., 1999). Finally, in studies of sex workers who have a high risk of exposure to *C. trachomatis* via *C. trachomatis-*infected clients, the probability of incident *C. trachomatis* infection correlates inversely with duration of prostitution (Brunham et al., 1996). Finally, immune dysfunction, as indicated by HIV seropositive status, is a risk factor for incident *C. trachomatis* infection (*ibid*).

Evidence for an association of IFNγ with genital chlamydial infection is supported by multiple experimental studies in animals (reviewed in detail in Rank and Whittum-Hudson, 2010) and observational/correlative studies in humans. Pertinently, in the murine model of genital infection using *Chlamydia muridarum*, a species that shares substantial genomic synteny with *C. trachomatis*, T-cells and IFNγ are critical to the resolution of, and subsequent protection from, genital infection (Ramsey et al., 1988; Cain and Rank, 1995; Su et al., 1997; Johansson and Lycke, 2001). Thus, CD4, MHC class II, IFNγ and IFNγ-receptor depletion/knockout results in chronic infection, uncontrolled *C. muridarum* bacterial burden and/or lack of protection from re-infection (Cotter et al., 1997; Perry et al., 1997; Morrison et al., 2000; Li et al., 2008; Jupelli et al., 2010; Andrew et al., 2013). Interestingly, IDO1 is poorly induced by IFNγ in murine epithelial cells, and is not required for resolution of genital *C. muridarum* infection (McClarty et al., 2007). Rather, mice restrict *C. muridarum* though a cell-autonomous resistance mechanism by a large family of IFNγ-inducible GTPases called immunity related GTPases (Nelson et al., 2005; Miyairi et al., 2007; Coers et al., 2008; Burian et al., 2010). Consistent with the molecular mechanisms underlying IFNγ-mediated restriction in mice differing from those operant in humans, subsequent elegant molecular and cross-species studies indicate the genes that are divergent in *C. muridarum* and *C. trachomatis* strongly correlate with the ability to evade species-specific IFNγ effector activities. Similarly comparisons of genital and ocular *C. trachomatis* serovars indicate a strong correlation with evasion of tissue-specific IFNγ effector activities (Morrison, 2003).

**Figure 1:**
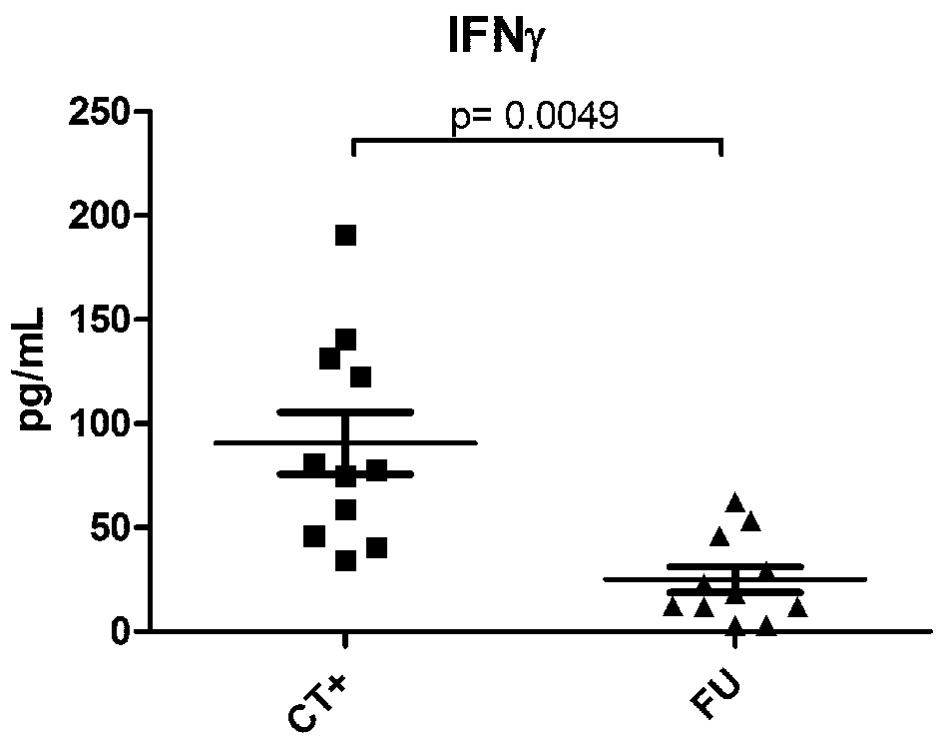
**IFNγ levels in paired endocervical secretion samples from women during, and post-antibiotic resolution, of *C. trachomatis* infection**. Endocervical secretions, harvested as previously described (Gumbi et al., 2008), were collected from 11 women during an ongoing NAAT+ *C. trachomatis* infection, and again 3–6 weeks after azithromycin treatment and a confirmed NAAT− *C. trachomatis* test. Samples were quantified using multiplex cytokine analysis (Millipore), and comparisons between infected and non-infected states investigated by a Wilcoxon-Mann-Whitney test. A *p*-value of < 0.05 was considered significant. CT+ indicates samples from patients with an active infection and FU indicates post-antibiotic follow-up samples.

In human genital infections, local cervical T-cell infiltrates and genital IFNγ concentrations are significantly elevated during active infection, higher in women with recurrent vs. primary infection, and decreased upon resolution of infection (Figure 1), and (Arno et al., 1990; Loomis and Starnbach, 2002; Agrawal et al., 2007; Ficarra et al., 2008; Sperling et al., 2013). Local IFNγ-producing *C. trachomatis-*specific CD4 T-cells are found in the endometrium of women with a high risk of exposure to *C. trachomatis* (Ondondo et al., 2009). Systemically, anti-chlamydial IFNγ-producing T-cells generally peak 1–2 months after active infection in most antibiotic-treated women (Vicetti et al., 2012); however, in highly-exposed women, IFNγ-producing T-cells that recognize epitopes from *C. trachomatis* HSP60 correlate with protection against incident *C. trachomatis* (Cohen et al., 2005). Finally, in highly exposed HIV seropositive women, a low CD4 count is associated with low *C. trachomatis-*induced IFNγ production (Cohen et al., 2000), and increased the risk of infection spread to the upper reproductive tract and PID (Kimani et al., 1996). While human research is challenging, we believe that new studies and tools are critical to further delineate, and mechanistically dissect, the complex relationships that exist between host immunity and bacteria in the context of the local genital environment.

## The human epithelial cell and *C. trachomatis* response to IFNγ and depletion of tryptophan

Because *C. trachomatis* is a tryptophan auxotroph, IDO1-mediated depletion of tryptophan curtails its growth (Taylor and Feng, 1991; Beatty et al., 1994a). *C. trachomatis* development is initiated when an infectious, but largely metabolically inactive, EB attaches to and enters the host cell. The internalized EB differentiates and replicates by binary fission as reticulate bodies (RBs) within a cytoplasmic membrane-bound inclusion. The completion of the developmental cycle is marked by re-differentiation of RBs into EBs that egress from the host cell. However, under conditions of limited tryptophan availability, the developmental cycle is arrested in the RB stage in a manner that results in morphologically distinct, large aberrant, viable but non-cultivable, persistent growth forms (Beatty et al., 1994a). Restoration of tryptophan availability can reactivate these persistent forms to return to normal development (Byrne et al., 1986). Similarly, provision of indole can reactivate persistent forms from genital, but not ocular, serovars, because the former synthesize a functional tryptophan synthase in response to tryptophan starvation (Caldwell et al., 2003).

## Chlamydial gene expression patterns induced by IFNγ

The induction of persistent forms with IFNγ, and their subsequent reactivation, have been studied *in vitro* through microarray analysis. Persistent growth, characterized by large aberrant RBs, led to the up-regulation of genes involved in tryptophan utilization, DNA repair and recombination, phospholipid biosynthesis and translation. Up-regulation of the repressible *trp*BA operon (Belland et al., 2003) confirms the previous observations that IFNγ treatment reduces intracellular concentrations of tryptophan. In addition, a number of early genes were up-regulated, particularly the *euo* gene (30-fold increase), which encodes a DNA-binding protein that has been shown to bind to a late gene promoter region (i.e., *omc*AB Zhang et al., 1998) to repress expression. Euo has been shown to down-regulate multiple late genes (Rosario and Tan, 2012); down-regulation of *C. trachomatis* genes involved in RB to EB differentiation, proteolysis and peptide transport, and cell division were seen during persistent growth induced by IFNγ. These transcriptional analyses were consistent with the biological properties associated with aberrant RBs in that the RBs were blocked in cytokinesis, and the developmental cycle was arrested at a point preceding late gene expression. Indeed, EM analyses of persistent forms induced by IFNγ treatment, indicates that large aberrant RBs are arrested in cytokinesis. This morphology appears to contrast quite strikingly with persistent forms induced by exposure to penicillins or the danger signal adenosine (Pettengill et al., 2009; Skilton et al., 2009), but resemble the atypical forms observed previously upon IFNγ treatment (Beatty et al., 1993, 1994b).

Recently, we have developed protocols that allowed the parallel assessment of both *C. trachomatis* gene transcription (specifically the *euo*:*omcB* ratio), and ultrastructure of *C. trachomatis* growth forms found *in vivo* in human endocervical infection (Lewis et al., 2014). The sampling protocols also permitted the quantification of indole and IFNγ levels from the same environment (*ibid*). Using this multi-parameter sampling approach, we were able to visualize two strikingly different growth patterns of *C. trachomatis* growth in two patients. Specifically, one infection was characterized by morphologically normal late-stage inclusions, high viable bacterial numbers, and a low *euo*:*omcB* mRNA expression profile, together with the presence of a high concentration of indole and no IFNγ. In contrast, the second infection included morphologically aberrant forms similar in ultrastructure to *in vitro* persistence induced by IFNγ, a high *euo*:*omcB* mRNA expression profile, low infectious titer, and large bacterial DNA load together with a local IFNγ response. This approach, that may be expanded to include an even more detailed analysis of *C. trachomatis* and host gene expression, indicate the distinct possibility of understanding how *C. trachomatis* grows in the genital tract, along with the environmental stresses, including IFNγ, which it encounters.

## Tryptophan supplementation reverses transcriptional changes induced by IFNγ

In the *in vitro* model of IFNγ-induced persistence, removal of IFNγ and supplementation with added tryptophan led to a rapid reactivation from persistent growth (Belland et al., 2003). During reactivation the expression differences rapidly returned to control levels, i.e., *euo* expression dropped 20-fold in 12 h. The transcriptional changes in the presence of IFNγ that result in persistent growth appear to constitute a persistence stimulon. Thus, we earlier postulated that this coordinated biological response is speculated to have evolved to allow the organism to rapidly respond to immunological pressure in a manner that allows for a period of resistance followed by rapid recovery after the waning of the host response.

## Genital serovars of *C. trachomatis* can synthesize a functional tryptophan synthase

Shaw *et al*, who reported that ocular serovars encoded a truncated TrpA protein (Shaw et al., 2000), initially described differences in tryptophan synthase between ocular and genital serovars of *C. trachomatis*. This analysis was extended by Fehlner-Gardiner et al., who reported that genital, but not ocular, *C. trachomatis* serovars encode a functional, tightly regulated, tryptophan synthase (*trpBA*) permitting the bacteria to synthesize tryptophan from indole (Fehlner-Gardiner et al., 2002; Akers and Tan, 2006; Carlson et al., 2006). Along with other genes that affect pathogenesis and distinguish ocular and genital serovars of *C. trachomatis* (Carlson et al., 2004; Nelson et al., 2006; Taylor et al., 2010), the enzymes required for the biosynthesis of tryptophan are also encoded by genes within the plasticity zone (McClarty et al., 2007). The complement of *trp* genes within this region varies between chlamydial species. *C. pneumoniae* and *C. muridarum* do not encode *trp* genes (Xie et al., 2002a). *C. psittaci* lacks the *trpE* gene and is therefore incapable of synthesizing the anthranilate, an essential precursor for tryptophan biosynthesis. *C. trachomatis* urogenital isolates only have a subset of *trp* genes, encoded in the plasticity zone: *trpR*, encoding a *trp* repressor; and *trpA* and *trpB*, encoding homologs of the α (TrpA) and β (TrpB) subunits of tryptophan synthase. As reviewed recently (Raboni et al., 2009; Miles, 2013), tryptophan synthase is a tetramer consisting of two α subunits and two β subunits (α2β2). Functional and sequence analyses indicate that in most bacterial species that express a α2β2 tetramer, the enzyme is predicted to be bi-functional and catalyze the cleavage of indole glycerol-3-phosphate (IGP) to indole and glyceraldehyde-3-phosphate (TrpA catalyzed α reaction), followed by the reaction of indole with serine to form tryptophan (TrpB catalyzed β-replacement reaction) (Xie et al., 2002b; Raboni et al., 2009; Miles, 2013). However, sequence analysis, and complementation studies conducted in *E. coli*, together indicate that the TrpA subunit in *C. trachomatis* appears to function structurally but not enzymatically (Fehlner-Gardiner et al., 2002; Xie et al., 2002b). As a consequence, the TrpA/TrpB tetramer expressed by *C. trachomatis* during tryptophan starvation cannot use IGP as a substrate for tryptophan biosynthesis; rather, it requires indole (Fehlner-Gardiner et al., 2002). Therefore, indole, and not IGP, may be an important molecule in chlamydial growth by modulating the effectiveness of the IFNγ response in bacterial clearance. Subsequent studies by Caldwell et al. verified the absolute correlation between tissue tropism and *trpBA* expression in >200 clinical isolates (Caldwell et al., 2003). All urogenital isolates had an intact *trpRBA* operon, while >90 ocular isolates invariably had frame-shift-inducing deletions within the *trpA* or *trpB* genes or had deleted the entire operon (*ibid*). This provided a molecular basis for the observation that only genital isolates could synthesize tryptophan from indole. While the enzymatic capacity of the chlamydial tryptophan synthase to salvage indole has been assessed only in *E. coli*, pioneering genetic studies reveal that a *C. trachomatis* null mutant in *trpB* has lost the capacity to escape IFNγ-mediated tryptophan depletion via indole salvage (Kari et al., 2011). Together, these data reveal a strong selective pressure for genital *C. trachomatis* strains to use indole salvage as a mechanism to escape IFNγ-mediated eradication by the host. Therefore, the ability to overcome tryptophan starvation may provide a mechanism through which *C. trachomatis* could cause extended and chronic infections in some women despite a robust induction of IFNγ. Critically, the availability of indole within the infection microenvironment is predicted to modulate the effect of IFNγ. Genital chlamydial infections occur in the context of the genital microbiome, of which the vaginal microbiome has been characterized in depth. The vaginal microbiome, particularly when perturbed, has been postulated to be the source of indole (Caldwell et al., 2003).

## An indole-poor ocular microenvironment may select against a functional tryptophan synthase

Although not the focus of this review, the stark contrast in the ability of urogenital and ocular *C. trachomatis* isolates to express a functional tryptophan synthase is intriguing to us. Unlike the vaginal microbiome, which as described below has been characterized under a variety of conditions, the normal ocular microbiome is far less characterized. The two most prominent phyla in the ocular microbiome from four healthy subjects were *Pseudomonas* and *Bradyrhizobium*, neither of which produce indole (Dong et al., 2011). We note that the absence of indole in the conjunctival microenvironment is insufficient to explain why ocular chlamydial isolates have uniformly lost the capacity to express a functional tryptophan synthase, most often as a consequence of point mutations. This apparent negative selection may result from an alternative enzymatic reaction catalyzed by tryptophan synthase when indole is absent; specifically, the β-elimination reaction in which L-serine is deaminated to produce pyruvate and ammonia (Kumagai and Miles, 1971; Miles and McPhie, 1974; Xie et al., 2002b; Raboni et al., 2009). While initially described as a function of β2 dimers, detailed studies examining the functions of the α2β2 tetrameric enzyme from *S. typhimurium* have revealed that it also catalyzes the β-elimination reaction necessary to produce ammonia (Ahmed et al., 1991; Raboni et al., 2005). While this catalysis is allosterically curtailed in the tetrameric enzyme by the glyceraldehyde-3-phosphate product of the α reaction, the *C. trachomatis* TrpA sequence, and functional analyses, indicate that it cannot bind IGP and catalyze the α reaction (Fehlner-Gardiner et al., 2002; Xie et al., 2002b). Further, sequence changes in the loop 6 of the *C. trachomatis* α subunit are predicted to prevent the inter-subunit interactions necessary for allosteric control (Schneider et al., 1998), consistent with the outcome of mutational analyses of the *S. typhimurium* enzyme (Yang and Miles, 1992; Brzovic et al., 1993; Kulik et al., 2002; Raboni et al., 2005). Pertinently, the active-site residues necessary for β-elimination catalysis to produce ammonia remain highly conserved in TrpB from *C. trachomatis*. Therefore, the α2β2 tetrameric enzyme from *C. trachomatis* is predicted to catalyze the generation of ammonia from serine when indole is absent. In addition to its anti-microbial effects (Rideal, 1895), ammonia is also known to induce apoptosis of epithelial cells that express the NMDA receptor (Suzuki et al., 2002; Sachs et al., 2011). In this context, the production of ammonia is currently proposed to underlie apoptosis of gastric epithelial cells induced by *H. pylori* (Seo et al., 2011). It may be of relevance that although their expression in the normal conjunctiva has not been examined, NMDA receptors are expressed in some ocular epithelial cell-lines (Oswald et al., 2012). As reviewed recently, cellular infiltrates associated with human conjunctival *C. trachomatis* infections include T-cells, which are capable of producing IFNγ (Hu et al., 2013). The consequential IFNγ-induced expression of a functional tryptophan synthase in an indole-limiting environment is predicted to generate ammonia by deaminating serine, with ensuing effects that may directly select against bacterial replication or cause apoptosis of infected cells prior to completion of the normal *C. trachomatis* developmental cycle.

Regardless of the pressure against the expression of a functional tryptophan synthase in trachoma, the strong selective pressure on urogenital isolates to retain the capacity to synthesize tryptophan via indole salvage is striking. It is likely this pressure reflects the influence of the urogenital microbiome on *C. trachomatis* replication in the face of a protective immune response.

## The vaginal microbiome and its effects

It is now well appreciated that the vaginal microbiome can significantly impact the reproductive health of women, their fetuses and their newborns (Hillier et al., 1995; Zhou et al., 2010; Ma et al., 2012). Bacterial vaginosis (BV), which affects 29% of reproductive age women in the US, is characterized by the loss of *Lactobacillus* species and a concomitant overgrowth of diverse anaerobes (Koumans et al., 2001, 2007). BV can be diagnosed by a gram stain-based scoring system termed the Nugent score and is calculated by assessing for the presence of large Gram-positive rods (chiefly *Lactobacillus* species), small Gram-variable rods and curved gram-variable rods; scores range from 1–10 and a Nugent of 7–10 is considered a diagnosis of BV and represents a sharp decrease in the number of Gram-positive rods with a simultaneous increase in the latter two morphotypes (Nugent et al., 1991; Delaney and Onderdonk, 2001). BV increases susceptibility to various STDs and PID (Haggerty et al., 2004) as well as the acquisition of HIV (Taha et al., 1998). Recent studies also indicate that the abnormal vaginal microbiome during BV affects the natural history of cervical human papillomavirus (HPV) and the development of cervical intraepithelial neoplasia (CIN) (Rodriguez-Cerdeira et al., 2012; Gao et al., 2013; Wheeler, 2013). Such observations indicate a dynamic relationship between the vaginal microbiome and the host; colonization by “normal” microbiomes may protect by preventing colonization by potential pathogens or by creating conditions that do not favor survival of the latter (Brotman et al., 2013). By disrupting this equilibrium, abnormal microbiomes create an environment that favors infection or colonization by various pathogens.

## Normal and BV-associated vaginal microbiomes

Advances in high-throughput sequencing have recently permitted the development of culture-independent methods to determine the composition of vaginal microbial communities (Ravel et al., 2011). They have also permitted the evaluation of the effect of various perturbations such as antibiotics, contraceptives and sexual activity on this microbiome (Gajer et al., 2012; Brotman et al., 2013; Ravel et al., 2013). Further, the altered microbiomes present in clinically defined conditions such as BV have been characterized (Ravel et al., 2011, 2013; Datcu et al., 2013). Cross-sectional surveys using such culture-independent methods have revealed the existence of several types of vaginal communities in normal, healthy, women with distinct bacterial species compositions (Ravel et al., 2011; Ma et al., 2012; Brotman et al., 2013). Four of these communities are dominated by *Lactobacillus* species: Type I—*L. crispatus*; Type II—*L. gasseri*; Type III—*L. iners*; and Type V—*L. jensenii*. The fifth community type, labeled Type IV, is dominated by facultative and strict anaerobes combined with insignificant numbers of lactobacilli. The Type IV community type is most closely associated with BV, as defined by a Nugent score of 7–10. A study examining the cervical microbiome has largely recapitulated the vaginal findings (Smith et al., 2012).

## Effects of the vaginal microbiome on *C. trachomatis*

Several studies indicate the vaginal microbiome can influence *C. trachomatis* infection. For example, pregnant women with flora in which H_2_O_2_-positive *Lactobacillus* spp. predominate are less likely to be infected by *C. trachomatis* (Hillier et al., 1992). In contrast, studies using an American cohort, aged 15–30, indicated a strong correlation between BV, as defined by Nugent scores ranging from 7–10, and *Chlamydia* infection (odds ratio 3.4) (Wiesenfeld et al., 2003). These results were recapitulated in studies examining a Japanese cohort of similarly aged women. The latter studies found an association between BV (NS 7–10) and *Chlamydia* infection with an odds ratio of 3.5 (Yoshimura et al., 2009).

Differences between the normal and BV microbiome could influence the normal development of *Chlamydia*, and the effect of IFNγ on normal development in multiple ways. First, we know that some members of the BV microbiome can express a functional tryptophanase to produce indole from tryptophan (Sasaki-Imamura et al., 2011), thus providing genital serovars of *C. trachomatis* with a means to obtain tryptophan via indole salvage. We also know from our recent studies that indole is present in the vaginal secretions of patients with BV (Lewis et al., 2014). Therefore, BV microbiome-produced indole could ameliorate the effect of IFNγ-induced tryptophan depletion on *Chlamydia* development. Indeed, tryptophan synthesis through indole salvage is likely to be desirable for *C. trachomatis* for several reasons. First, under hypoxic conditions, IDO1 can catabolize tryptophan but not indole. Second, IDO1-mediated catabolism depletes tryptophan levels within the chlamydial inclusion by decreasing extracellular and cytoplasmic tryptophan, and not by directly acting upon tryptophan within the inclusion. Third, mammalian cells lack a tryptophan synthase activity; therefore, tryptophan biosynthesis through indole salvage occurs solely within the chlamydial inclusion, providing only the bacterium with this essential amino acid with no competition from the host cell. Finally, because IDO1 catabolizes only extracellular and cytoplasmic tryptophan, it will not affect tryptophan synthesized within the inclusion.

The BV microbiome might also limit the effect of IFNγ on *C. trachomatis* by other mechanisms. The *Lactobacillus* sp. that predominate in vaginal microbiome types I, II, III, and V do not synthesize indole. In addition, they create a highly acidic H_2_O_2_-rich environment (pH < 4.2) that is also not conducive to chlamydial growth and development (Das et al., 2005; Haggerty et al., 2009). In contrast, anaerobes present during BV (vaginal microbiome type IV) raise the pH to >4.6 and simultaneously produce a hypoxic microenvironment. It is pertinent to note that the normal vaginal pH ranges from 3.8 to 4.5. Chlamydial re-infection is favored at higher pH, and hypoxia reduces the restrictive effect of IFNγ on chlamydial growth in two ways: (1) Low oxygen partial pressure conditions (pO_2_) limit both IFNγ-dependent signaling pathways (Roth et al., 2010); and (2) The enzymatic capacity of IDO1 to catabolize tryptophan by dioxygenation is significantly hampered by low pO_2_ (Herbert et al., 2011).

## Possible sources of indole in the genital tract

Although the normal and BV microbiomes display variations between patients, there are several commonalities in their composition. The normal vagina (Nugent scores 0–3) contains ~10^7^ bacteria/10 ng of DNA recovered from vaginal swabs, in which *Lactobacillus* spp. predominate. During BV (Nugent score 7–10), the bacterial load is increased to ~10^9^ bacteria/10 ng DNA, in which combinations of *Prevotella* spp. (>10^8^/10 ng), *Gardnerella vaginalis* (10^7^/10 ng), *Atopobium vaginae* (10^5^/10 ng), and/or *Megasphaera* spp. (10^7^/10 ng) become abundant. A large increase in the fastidious bacterium of the order *Clostridiales* (BVAB1—10^8^/10 ng) is also observed. *Lactobacillus* spp. do not produce indole, however many *Prevotella* spp. and strains can express a tryptophanase (*tnaA*) to produce indole (Sasaki-Imamura et al., 2011). Many sequenced members of the order *Clostridiales* also encode a tryptophanase gene. BV infections are occasionally coincident with infections by the protozoan pathogen *Trichomonas vaginalis* that can also express a tryptophanase to produce indole (Lloyd et al., 1991; Zubacova et al., 2011). Thus *T. vaginalis* co-infections may represent another mechanism by which vaginal co-infections impact the effect of IFNγ on *C. trachomatis.*

These results clearly indicate that indole-producing bacteria are present within patients, with the levels of indole increasing with higher Nugent scores. Several recent studies examining the BV microbiome indicate that the increased representation of *Prevotella* spp. is the strongest correlate of BV (NS 7–10) (Datcu et al., 2013, 2014). Consistent with these, a metagenomic study examining mRNA expressed by the BV microbiome indicated that *Prevotella* spp. mRNA accounted for approximately 30% of all the mRNA expressed by the BV microbiome (Twin et al., 2013). The high representation of *Prevotella* spp. mRNA, coupled with the observation that some oral strains of *Prevotella* can express a tryptophanase, led us to test whether genital isolates of *Prevotella* from BV patients could also synthesize indole. For this, residual speculum fluid from three BV patients was used to isolate anaerobes using laked blood agar kanamycin/vancomycin media. Kanamycin/vancomycin-resistant constitutive anaerobes were isolated from all three samples and tested for indole production. Multiple isolates from two patients produced indole robustly, while only a single isolate from the third patient did. Partial 16S rRNA sequences (GenBank accession numbers KJ435311—KJ435324) confirmed all but one of the isolates (indole producing and non-producing) to be *Prevotella* spp. These results indicate that indole-producing bacteria can be found in the microbiome from the female genital tract. Further, there are variations between patients, such that even within the same genus (i.e., *Prevotella*), genetic differences between isolates can alter indole availability in a patient-specific manner.

## A general role for indole availability in the genital tract

Genomic and functional analyses of other bacteria indicate that indole is available in the genital tract. For example, while environmental and nosocomial infection-associated isolates of *Staphylococcus aureus* retain the capacity to synthesize tryptophan *de novo* from chorismic acid, 80% of the Toxic Shock Syndrome Toxin (TSST) producing *S. aureus* isolates have lost this capacity, typically due to a deletion within the *trpD* gene (McGavin et al., 2012). However, these TSS-associated isolates continue to encode a functional tryptophan synthase, suggesting that indole and/or IGP is likely available within the genital microenvironment, permitting bacterial growth even during tryptophan starvation (*ibid*).

Similarly, several members of the normal and BV microbiome can synthesize tryptophan *de novo* from chorismic acid, others, such as *G. vaginalis*, resemble CT, in that they can only synthesize tryptophan by indole salvage. An analysis of the *Gardnerella vaginalis* reference genome (GenBank accession NC_013721) indicates it encodes a TrpB subunit that is 54% identical to the TrpB subunit from *Escherichia coli*, with no additional domains. However, no sequenced isolate of *G. vaginalis* encodes a TrpA subunit, implying that akin to CT, *Gardnerella* can use indole, but not IGP, in the environment to synthesize tryptophan. Therefore, it is likely that during tryptophan starvation, *C. trachomatis* and *Gardnerella* both rely on indole produced by tryptophan autotrophs that also encode a tryptophanase. For this reason, the development and use of small molecule therapeutics that target tryptophanase will not only promote IFNγ-mediated *C. trachomatis* clearance, but also aid in the clearance of indole-dependent BV bacteria such as *G. vaginalis*.

## Concluding remarks

In this review, we have described the mechanism by which IFNγ could act as a protective cytokine against chlamydial infections. IFNγ's protective effects result from catabolism of the essential amino-acid tryptophan by the enzyme IDO1. Depletion of tryptophan can induce a viable but not cultivable persistent growth phenotype in *Chlamydia* that can be reactivated when the IFNγ-response wanes and/or tryptophan is made available in the environment. Consistent with this, IFNγ exposure induces a chlamydial pattern of gene expression that causes an up-regulation of genes that can synthesize tryptophan through indole salvage and a down-regulation of genes necessary for later stages of the chlamydial normal development cycle. Genomic analyses indicate that a selective pressure to maintain tryptophan synthesis via indole salvage has been applied strictly to every genital *Chlamydia* serovar. In contrast, no ocular serovars can salvage indole. Given that the genes necessary for indole salvage (*trp*BA) are present in a genetic plasticity zone, it is likely that indole availability in the infection microenvironment has been the selective factor to maintain the capacity to salvage indole. Consistent with this, we have found indole in vaginal secretions from patients that have BV, and have isolated indole-producing bacteria from patients that have BV. Therefore, it is likely that natural immunity against chlamydial infections driven by a protective IFNγ response will be attenuated in patients that have BV, dependent on the bacterial representation within individual patients. For these reasons, further studies that examine the correlates between spontaneous clearance of chlamydial infections, the host cytokine response, the host microbiome, and metabolites such as indole, are essential for the successful development of a protective vaccine against *Chlamydia*. Further, understanding the nature and contribution of BV-associated bacteria and their indole-producing capacity and how this relates to the effectiveness of an IFNγ-mediated resolution of *C. trachomatis* infection may also guide the management of BV in *C. trachomatis-*infected patients in the future.

### Conflict of interest statement

The authors declare that the research was conducted in the absence of any commercial or financial relationships that could be construed as a potential conflict of interest.
